# Serum progesterone and vaginal gel dose in artificial-cycle frozen embryo transfer: association with pregnancy outcome

**DOI:** 10.3389/fendo.2026.1717071

**Published:** 2026-03-05

**Authors:** Hsiao-Chin Huang, Yu-Li Chuang, Chueh-Ko Yang, Horng-Der Tsai, Cheng-Hsuan Wu, Yu-Jing Chen, Hsin-Hung Wu

**Affiliations:** 1 Department of Obstetrics and Gynecology, Changhua Christian Hospital, Changhua, Taiwan; 2 Department of Changhua Christian Hospital Women’s Health Research Laboratory, Changhua, Taiwan; 3 Department of Post-Baccalaureate Medicine, College of Medicine, National Chung Hsing University, Taichung, Taiwan

**Keywords:** artificial cycle, frozen embryo transfer, luteal phase support, serum progesterone, vaginal progesterone gel

## Abstract

**Purpose:**

To evaluate the clinical relevance of serum progesterone levels on the day of frozen embryo transfer (FET) and the dose of vaginal progesterone gel (Crinone) used for early luteal phase support (LPS) in artificial-cycle frozen embryo transfer (AC-FET).

**Methods:**

This single-center retrospective study analyzed 342 AC-FET cycles in patients aged 18–45 at Changhua Christian Hospital, Taiwan (April 2018–December 2022). Women aged 18–45 years undergoing AC-FET were included; cycles with >3 prior failed FETs, major comorbidities, donor oocytes, or missing outcome data were excluded. Pregnancy outcomes were assessed per cycle. Crinone (90 mg or 180 mg daily) was administered for 5 days after endometrial thickness ≥7 mm and progesterone <1.5 ng/mL. Serum progesterone was measured on the day of FET. Clinical pregnancy rates based on gestational sac (CPR-S), fetal heartbeat (CPR-H), and live birth rate (LBR) were evaluated. Receiver operating characteristic (ROC) evaluated the predictive value of serum progesterone levels. Associations between study variables and pregnancy outcomes were assessed using univariate and multivariable logistic regression. Comparisons of pregnancy outcomes between the high- and low-dose Crinone groups were further conducted using inverse probability of treatment weighting (IPTW) to adjust for baseline differences between dosage groups.

**Results:**

Serum progesterone levels demonstrated poor predictive performance for CPR-S, CPR-H, and LBR (AUC range: 0.541–0.559) and were not independently associated with pregnancy outcomes in multivariable analyses. After adjustment using inverse probability of treatment weighting, a higher Crinone dose (180 mg vs. 90 mg) was significantly associated with higher CPR-S (OR, 1.87; 95% CI, 1.11–3.17; p = 0.020), CPR-H (OR, 2.11; 95% CI, 1.24–3.59; p = 0.006), and LBR (OR, 2.10; 95% CI, 1.31–3.38; p = 0.002).

**Conclusion:**

Serum progesterone levels measured on the day of FET did not predict pregnancy outcomes in AC-FET cycles using vaginal progesterone gel. A higher Crinone dose was associated with higher early clinical pregnancy rates compared with a lower dose.

## Highlights

- Serum progesterone levels measured on the day of frozen embryo transfer do not predict pregnancy outcomes in artificial-cycle FET using vaginal progesterone gel for early luteal phase support.
- A higher Crinone dose (180 mg) was associated with higher clinical pregnancy rates compared with a lower dose (90 mg).

## Background

Frozen embryo transfer (FET) is being used more frequently in assisted reproductive technology (ART), with a corresponding rise in live births attributed to FET in the US (1). FET accounted for 41% of ART cycles in the US in 2021 and 31.2% of ART cycles in Europe in 2019 (2, 3). FET is also widely utilized in Taiwan, accounting for 19,614 FET cycles out of 49,652 ART cycles (39.5%) in 2021 (4, 5). This growing utilization and interest underscore the importance of optimizing endometrial preparation methods for FET to improve pregnancy outcomes.

Of the different types of endometrial preparations widely used for FET, artificial cycles (AC) facilitated by hormone replacement treatment (HRT) offer the most flexibility and convenience in FET scheduling (6, 7). AC-FET requires exogenous endocrine support, including supplemental estrogen for endometrial growth and progesterone for endometrial differentiation, also known as luteal phase support (LPS) (8). Serum levels of progesterone are often monitored to optimize LPS, aiming to improve implantation, pregnancy, and delivery success rate (6, 8, 9). Several studies have reported that in AC-FET protocols, serum progesterone levels <9-10ng/mL on the day or the day before FET adversely affect live birth and ongoing pregnancy rates (10). However, defining a serum progesterone threshold predictive of FET success is challenging, not only due to differences in patient characteristics, but also due to variations in progesterone pharmacokinetics across different administration routes. Despite ongoing research into the optimal route, dose, and timing of progesterone supplementation for LPS, the optimal serum progesterone level for successful pregnancy outcomes in AC-FET cycles has yet to be established.

Among the various progesterone administration routes and regimens in use - including intramuscular, oral, subcutaneous, and vaginal – vaginal progesterone is generally preferred for its convenience and high patient acceptance (7, 11). Compared with oral progesterone, vaginal administration avoids extensive first-pass hepatic metabolism, provides higher local endometrial exposure, and is associated with fewer systemic adverse effects. Crinone, a vaginal progesterone gel, provides sustained hormone release with lower serum concentrations and is commonly used in clinical practice (12). Serum progesterone thresholds have been proposed in AC- FET protocols using oral or intramuscular progesterone, whereas findings have been inconsistent for vaginal progesterone regimens (13, 14). Evidence specific to Crinone-based protocols remains limited, and due to preferential uterine uptake and variable systemic absorption, serum progesterone levels may not reliably reflect endometrial exposure in these cycles. Previous studies have suggested that increasing the dose of vaginal progesterone supplementation may improve pregnancy outcomes in FET cycles, including studies specifically evaluating progesterone gel (Crinone) and other transvaginal formulations (15, 16). However, the clinical relevance of serum progesterone levels in Crinone-based AC-FET cycles remains unclear.

We hypothesized that serum progesterone levels on the day of FET would be associated with pregnancy outcomes and that higher vaginal progesterone gel dosing would be associated with higher clinical pregnancy rates. Accordingly, this study aimed to evaluate the association between serum progesterone levels on the day of embryo transfer (ET) and clinical pregnancy outcomes in AC-FET cycles using vaginal progesterone gel for early LPS. Additionally, pregnancy outcomes were compared between two AC-FET regimens employing different doses of vaginal progesterone gel.

## Materials and methods

### Study design and patients

We retrospectively analyzed FET cycles performed at a tertiary reproductive medical center in central Taiwan between April 2018 and December 2022. Eligible patients included women aged 18 to 45 years who underwent IVF therapy and were scheduled for an FET procedure. In preparation for FET, the endometrium was conditioned using HRT, which included estradiol or Premarin. Following 12–14 days of exogenous hormone administration and confirmation of an endometrial thickness ≥ 7 mm, vaginal progesterone gel (Crinone^®^, 8%) was administered daily for early LPS.

We excluded women aged >45 years, those with >3 failed FET attempts (to reduce potential heterogeneity related to recurrent implantation failure), major comorbidities (e.g., hypertension or diabetes) (to establish a relatively healthy cohort and minimize confounding), transfer of more than two embryos, or missing laboratory or outcome data. Cycles involving donor oocytes, endometrial thickness below 7 mm, intolerance to Crinone, or nonadherence or intolerance to luteal phase supplementation were also excluded. Lastly, embryos screened via preimplantation genetic screening (PGS) were not included. This was because the additional cost led to a low number of cases, and exclusion helped minimize bias.

This study was approved by the Institutional Review Board of Changhua Christian Hospital prior to initiation (IRB Number: 240128), with a waiver of written informed consent. The study was performed according to the principles of the Declaration of Helsinki.

### AC-FET treatment protocol

Patients undergoing AC-FET underwent endometrial preparation with HRT, consisting of estradiol or conjugated estrogen (Premarin^®^) for 12–14 days. Transvaginal ultrasound was performed afterwards, and once the endometrium thickness reached ≥ 7 mm and serum progesterone level was < 1.5 ng/ml, LPS was initiated using vaginal progesterone gel, administered once or twice daily (90 mg or 180 mg/day) for 5 days. This progesterone threshold was used to exclude premature progesterone elevation and ensure appropriate endometrial–embryo synchronization, consistent with commonly adopted programmed FET protocols (17). FET was performed on the sixth day after LPS initiation, and serum progesterone level was measured on the day of FET.

After FET, all patients received additional progesterone supplementation (intramuscular and/or oral) according to institutional practice.

### Serum progesterone measurement

Serum progesterone level was measured on the day of FET (i.e., prior to embryo transfer). Blood samples were collected in the morning (09:00–11:00) under non-fasting conditions. Progesterone was quantified using a chemiluminescent immunoassay (Access Progesterone assay, Beckman Coulter), with an analytical sensitivity of 0.10 ng/mL and a measurement range of 0.10–40.0 ng/mL.

### Reproductive outcomes

The outcomes evaluated included the clinical pregnancy rate, defined by the presence of a gestational sac (CPR-S) or detection of a fetal heartbeat via ultrasound (CPR-H); the live birth rate (LBR); and the biochemical pregnancy rate, defined as serum β-hCG ≥ 10 mIU/mL at 11–14 days post-FET.

### Other variables

Demographic, clinical, and embryological data were collected, including age (years), body mass index (BMI, kg/m²), anti-Müllerian hormone (AMH, ng/mL) levels, and endometrial thickness (cm) on the day of ET. Embryo quality was categorized using an adaptation of the Gardner blastocyst grading system, classifying embryos as high (3–6 AA, 4–6 AB, and 4–6 BA), mid (any BB, 1–3 AB, 1–3 BA, and 1–2 AA), or low (any AC, CA, BC, CB, and CC), based on overall blastocyst morphology (18). When two embryos were transferred, classification was based on the higher-grade embryo.

Reproductive history included the number of previous pregnancies, births, and miscarriages (<2 or ≥2). The causes of infertility were classified as anovulation, endometriosis, male factor, multiple factors, poor ovarian response (POR), tubal factor, unexplained infertility, or other causes. The number of embryos transferred (1 or 2) was also included.

### Late luteal phase support

After transfer, late LPS was continued according to institutional clinical practice, using various progesterone formulations and routes. The distribution of late LPS regimens according to Crinone dose is summarized in **Table 1**. These regimens were not protocolized based on serum progesterone levels and were not the primary focus of the present analysis (**Table 1**).

**Table 1:** Demographic and clinical characteristics.

	Total N=342	
	Mean	SD
Age, years	34.49	4.22
BMI, kg/m^2^	23.23	4.36
AMH, ng/mL	4.96	3.79
Endometrial thickness, cm	1.05	0.23
	n	%
Blastocyst morphological classification		
High	143	41.8%
Mid	68	20.0%
Low	131	38.3%
Previous pregnancies		
<2	280	81.9%
≥2	62	18.1%
Previous live births		
<2	331	96.9%
≥2	11	18.1%
Previous miscarriages		
<2	305	89.2%
≥2	37	10.8%
Cause of infertility		
Anovulation	110	32.2%
Endometriosis	21	6.1%
Male factor	38	11.1%
Multiple	28	8.2%
POR	37	10.8%
Tubal factor	29	8.5%
Unexplained	58	17.0%
Other	21	6.1%
Embryo transfer number		
1	134	39.2%
2	208	60.8%

SD, standard deviation; BMI, body mass index; AMH, anti-mullerian hormone; POR, poor ovarian response.

### Statistical analysis

Continuous variables are presented as means and standard deviations, and comparisons between dose groups were conducted using the Student’s *t*-test or the Mann-Whitney U test, as appropriate. Categorical variables are presented as counts and percentages. Comparisons between groups were performed using the Pearson chi-square test or Fisher’s exact test. To assess the predictive value of serum progesterone levels on the day of FET for pregnancy outcomes in cycles using Crinone for early LPS, receiver operating characteristic (ROC) curve analysis was performed, with the area under the curve (AUC) calculated as a measure of predictive performance. Univariate and multivariable logistic regression analyses were performed to determine the factors associated with the three outcomes: CPR-S, CPR-H, and LBR. Variables with a p<0.05 in univariate analyses were included in the multivariable model. The results are presented as odds ratios (ORs) with corresponding 95% confidence intervals (CIs).

To evaluate associations between vaginal progesterone gel dose and clinical pregnancy outcomes, the stabilized inverse probability of treatment weighting (IPTW) method was applied to address potential confounding by baseline covariates (19–21). Propensity scores were first estimated using baseline covariates associated with treatment assignment. Covariates were selected *a priori* based on clinical relevance and prior literature, including age, BMI, AMH, endometrial thickness, blastocyst morphological classification, reproductive history, and number of embryos transferred. Stabilized inverse probability weights were then calculated to create an IPTW-weighted pseudo-population with balanced baseline characteristics. Covariate balance before and after weighting was assessed using standardized mean differences (SMDs), with values <0.10 indicating adequate balance (19). Finally, weighted logistic regression models were applied within this IPTW-weighted cohort to estimate the association between vaginal progesterone gel dose and pregnancy outcomes.

A two-sided p-value <0.05 was considered statistically significant. All statistical analyses were performed using IBM SPSS statistical software version 22 for Windows (SPSS Inc., an IBM Company, Chicago, IL) and SAS software version 9.4 (SAS Institute Inc., Cary, NC, USA).

## Results

### Demographic and clinical characteristics

**Figure 1:**
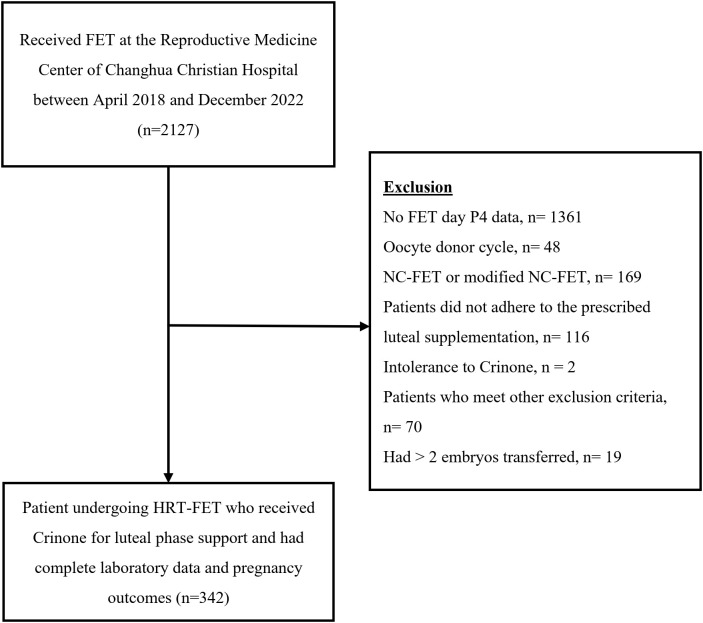
Flow chart of patient selection process.

A total of 342 cycles were included for analysis (108 cycles with Crinone 90 mg/day and 234 cycles with Crinone 180 mg/day) (**Figure 1**). The demographic and clinical characteristics were summarized in **Table 1**. The mean age of the participants was 34.5 years (standard deviation [SD]=4.2). There were 41.8% of participants having high-quality morphological embryos, and 18.1%, 18.1%, and 10.8% of participants had experienced at least two pregnancies, live births, or miscarriages. Most participants had two embryos transferred (n=208, 60.8%) (**Table 1**).

### Predictive performance of serum progesterone levels for pregnancy outcomes

**Figure 2:**
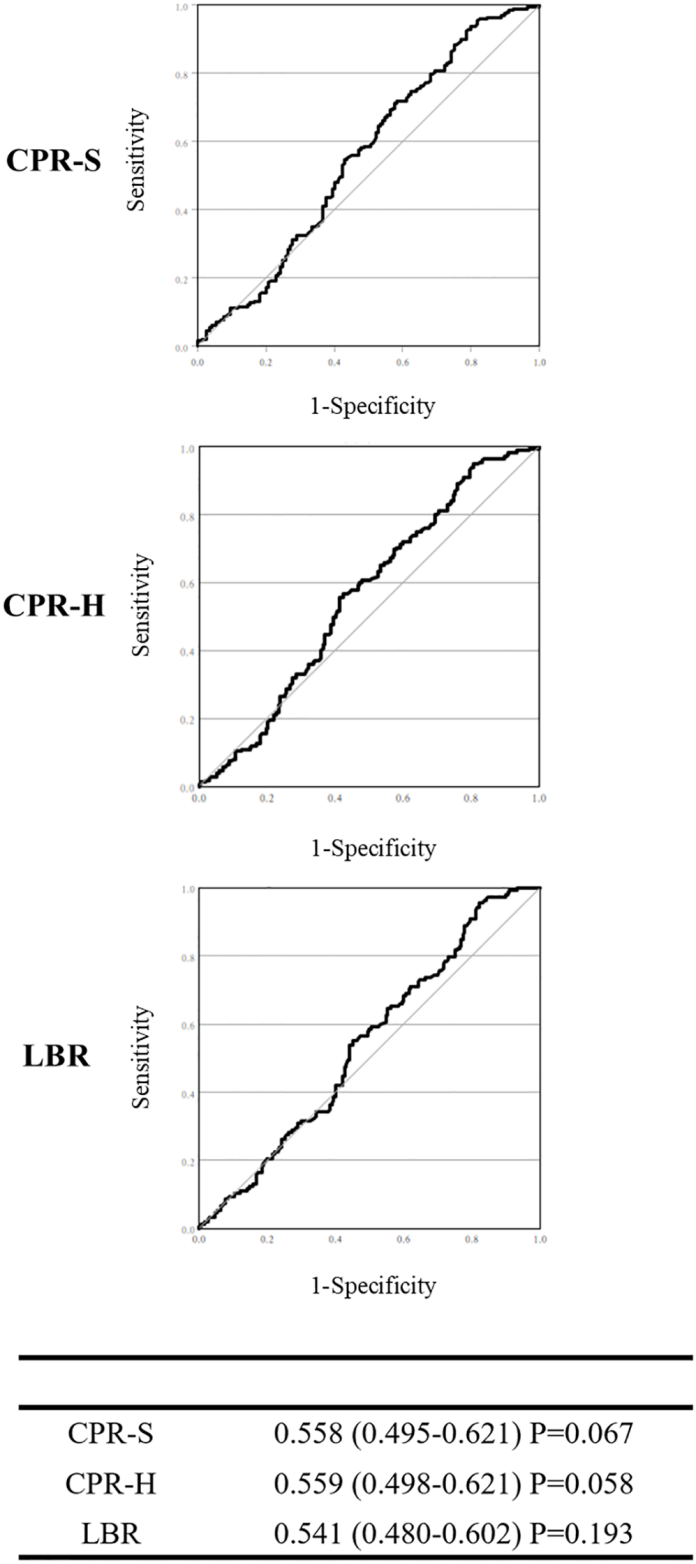
Predictive value of serum progesterone concentration on the day of frozen embryo transfer for clinical pregnancy (CPR-S, CPR-H, LBR) when Crinone was used for early luteal phase support.

The results of the ROC curve analyses are depicted in **Figure 2**. The AUC for serum progesterone levels on the day of FET in predicting CPR-S, CPR-H, and LBR ranged from 0.541 to 0.559, indicating poor predictive performance (**Figure 2**).

### Factors associated with pregnancy outcomes

The results of univariate and multivariable analyses on the factors associated with clinical pregnancy outcomes are summarized in **Table 2**. Serum progesterone levels were not significantly associated with any clinical pregnancy outcomes (CPR-S: OR, 1.02; 95% CI, 0.94–1.09; p = 0.685; CPR-H: OR, 1.00; 95% CI, 0.93–1.08; p = 0.959; LBR: OR, 1.04; 95% CI, 0.94–1.08; p = 0.846) after adjustment.

**Table 2:** Factors associated with clinical pregnancy outcomes.

	CPR-S				CPR-H				LBR			
	Univariate		Multivariate		Univariate		Multivariate		Univariate		Multivariate	
	OR (95%CI)	*P-value*	aOR (95%CI)	*P-value*	OR (95%CI)	*P-value*	aOR (95%CI)	*P-value*	OR (95%CI)	*P-value*	aOR (95%CI)	*P-value*
Serum progesterone level, ng/mL	1.05 (0.98–1.13)	0.137	1.02 (0.94–1.09)	0.685	1.04 (0.98–1.11)	0.194	1.00 (0.93–1.08)	0.959	1.04 (0.97–1.11)	0.288	1.01 (0.94–1.08)	0.846
Crinone dose												
180 vs. 90 mg	1.79 (1.13–2.83)	0.014*	1.82 (1.06–3.13)	0.031*	1.96 (1.23–3.11)	0.005*	2.11 (1.22–3.64)	0.008*	1.56 (0.98–2.49)	0.062	1.56 (0.91–2.69)	0.105
Age, years	0.99 (0.94–1.04)	0.676			1.00 (0.95–1.05)	0.981			0.98 (0.93–1.03)	0.370		
BMI, kg/m2	1.03 (0.98–1.08)	0.306			1.01 (0.96–1.06)	0.717			0.98 (0.94–1.03)	0.489		
AMH, ng/mL	1.05 (0.99–1.12)	0.107			1.05 (0.99–1.12)	0.106			1.01 (0.96–1.08)	0.635		
Endometrial thickness, cm	1.74 (0.66–4.57)	0.263			1.22 (0.48–3.11)	0.672			1.26 (0.50–3.22)	0.624		
Blastocyst morphological classification												
High (vs. low)	4.56 (2.46–8.44)	<0.001*	3.49 (1.83–6.65)	<0.001*	4.65 (2.48–8.74)	<0.001*	3.49 (1.80–6.77)	<0.001*	4.75 (2.45–9.21)	<0.001*	3.80 (1.92–7.53)	<0.001*
Mid (vs. low)	2.24 (1.22–4.10)	0.009*	1.99 (1.06–3.75)	0.033*	2.46 (1.31–4.63)	0.005*	2.23 (1.14–4.34)	0.019*	2.56 (1.31–5.00)	0.006*	2.32 (1.16–4.61)	0.017*
Previous pregnancies (vs. <2)	0.38 (0.22–0.68)	0.001*	0.54 (0.21–1.36)	0.190	0.35 (0.19–0.63)	<0.001*	0.40 (0.12–1.32)	0.134	0.45 (0.25–0.81)	0.008*	0.60 (0.32–1.12)	0.110
Previous live births (vs. <2)	0.40 (0.12–1.41)	0.154			0.20 (0.04–0.95)	0.043*	0.79 (0.11–5.45)	0.806	0.27 (0.06–1.26)	0.095		
Previous miscarriages (vs. <2)	0.45 (0.23–0.91)	0.026*	0.95 (0.31–2.91)	0.927	0.48 (0.23–0.97)	0.042*	1.34 (0.35–5.11)	0.665	0.57 (0.27–1.17)	0.123		
Embryo transfer number (vs. 1)	2.19 (1.41–3.42)	0.001*	2.36 (1.44–3.86)	<0.001*	2.17 (1.39–3.38)	0.001*	2.37 (1.44–3.90)	<0.001*	2.10 (1.34–3.29)	0.001*	2.14 (1.31–3.48)	0.002*

CPR, clinical pregnancy rate; H, heartbeat; LBR, live birth rate; S, sac; OR, odds ratio; aOR, adjusted odds ratio; CI, confidence interval; BMI, body mass index; AMH, anti-mullerian hormone.

*P < 0.05 indicates statistical significance.

After adjustment, Crinone dose (180 vs. 90 mg; OR, 1.82; 95% CI, 1.06–3.13; p = 0.031) and the number of embryos transferred (2 vs. 1; OR, 2.36; 95% CI, 1.44–3.86; p < 0.001) were significantly associated with an increasing odds of achieving CPR-S. Blastocyst quality was also significantly associated with CPR-S, with higher quality embryos increasing the likelihood of CPR-S (OR, 1.99; 95% CI, 1.06–3.75 for mid vs. low; OR, 3.49; 95% CI, 1.83–6.65 for high vs. low). Other variables, previous pregnancies and miscarriages, were not significantly associated with CPR-S (p > 0.05).

Factors significantly associated with higher CPR-H included Crinone dose (180 vs. 90 mg; OR, 2.11; 95% CI, 1.22–3.64; p = 0.008), blastocyst quality (OR, 2.23; 95% CI, 1.14–4.34 for mid vs. low; OR, 3.49; 95% CI, 1.80–6.77 for high vs. low), and number of embryos transferred (2 vs. 1; OR, 2.37; 95% CI, 1.44–3.90; p < 0.001). Previous pregnancy history, including the number of prior pregnancies, births, and miscarriages, was not significantly associated with CPR-H. (*p* > 0.05).

For LBR, the Crinone dose did not reach statistical significance (OR: 1.56; 95% CI: 0.91–2.69; p = 0.105). In contrast, higher blastocyst quality was associated with an increased likelihood of LBR (OR: 2.32; 95% CI: 1.16–4.61 for mid vs. low, and OR: 3.80; 95% CI: 1.92–7.53 for high vs. low). Additionally, transferring two embryos versus one was significantly associated with a higher LBR (OR: 2.14; 95% CI: 1.31–3.48; p = 0.002). The number of previous pregnancies was not significantly associated with LBR (p = 0.110) (**Table 2**).

### Comparative analysis of high versus low Crinone dose on pregnancy outcomes

Characteristics of the two dosage groups of vaginal progesterone gel are summarized in **Table 3**. The mean age of patients receiving progesterone 90 mg was 33.9 ± 3.6 years, compared to 34.8 ± 4.5 years in the 180 mg group. At the initiation of LPS with progesterone, the mean endometrial thickness was 1.1 ± 0.3 cm in the 90 mg group and 1.0 ± 0.2 cm in the 180 mg group. Except for age and previous live births, the distribution of characteristics between the dosage groups was significantly different (p < 0.05) (**Table 3**).

**Table 3:** Demographic and clinical characteristics by vaginal progesterone gel (Crinone) dose before and after IPTW.

	Before IPTW			After IPTW		
	90 mg	180 mg	SMD ^†^	90 mg	180 mg	SMD ^†^
	n=108	n=234		n=113	n=233	
	Mean ± SD			Mean ± SD		
Age, years	33.88 ± 3.55	34.76 ± 4.47	0.219	34.83 ± 3.62	34.56 ± 4.53	0.066
BMI, kg/m^2^	22.24 ± 3.83	23.69 ± 4.52	0.348	23.21 ± 5.16	23.26 ± 4.26	0.010
Endometrial thickness, cm	1.12 ± 0.25	1.01 ± 0.21	0.474	1.07 ± 0.21	1.06 ± 0.24	0.039
	n (%)			n (%)		
Blastocyst morphological classification			0.501			0.072
High	28 (25.9%)	115 (49.1%)		47 (41.1%)	98 (41.9%)	
Mid	55 (50.9%)	76 (32.5%)		26 (23.4%)	48 (20.5%)	
Low	25 (23.1%)	43 (18.4%)		40 (35.5%)	87 (37.6%)	
Previous pregnancies			0.287			0.121
<2	80 (74.1%)	200 (85.5%)		88 (77.9%)	193 (82.7%)	
≥2	28 (25.9%)	34 (14.5%)		25 (22.1%)	40 (17.3%)	
Previous live births			0.037			0.159
<2	105 (97.2%)	226 (96.6%)		106 (93.1%)	225 (96.6%)	
≥2	3 (2.8%)	8 (3.4%)		7 (6.9%)	8 (3.4%)	
Previous miscarriages			0.222			0.042
<2	91 (84.3%)	214 (91.5%)		101 (88.7%)	210 (90.0%)	
≥2	17 (15.7%)	20 (8.5%)		12 (11.3%)	23 (10.0%)	
Embryo transfer number			0.507			0.167
1	25 (23.1%)	109 (46.6%)		54 (47.6%)	92 (39.4%)	
2	83 (76.9%)	125 (53.4%)		59 (52.4%)	141 (60.6%)	

IPTW, inverse probability of treatment weighting; SD, standard deviation; BMI, body mass index.

^†^Absolute standardized mean difference (SMD) was presented. Values less than 0.1 indicate adequate balance between groups.

CPR-S and CPR-H were higher in the 180 mg group than the 90 mg group (62.4% vs. 48.1% and 56.4% vs. 39.8%, both p <0.05). No statistically significant differences in LBR and biochemical pregnancy rate were observed between the two Crinone dose groups, although a trend toward higher rates was noted in the 180 mg group (**Table 4**).

**Table 4:** Comparison of outcomes between 90 mg and 180 mg vaginal progesterone gel for early LPS in FET cycles.

	90 mg	180 mg	*P*-value^†^
	N=108	N=234	
	n (%)		
Biochemical pregnancy rate^‡^	72 (66.7%)	165 (70.5%)	0.474
CPR-S	52 (48.1%)	146 (62.4%)	0.013^*^
CPR-H	43 (39.8%)	132 (56.4%)	0.004^*^
LBR	40 (37.0%)	112 (47.9%)	0.061

LPS, luteal phase support; FET, frozen embryo transfer; CPR, clinical pregnancy rate; S, sac; H, heartbeat; LBR, live birth rate.

^†^Mann-Whitney U test for continuous variables and Fisher’s Exact test for categorical variables.

^‡^Biochemical pregnancy defined as β-hCG ≥ 10mIU/mL.

*P < 0.05 indicates statistical significance.

After IPTW weighting, adequate balance of most baseline covariates was achieved between Crinone dose groups (**Table 3**). Although the between-group SMDs of previous pregnancies (SMD = 0.121), previous live birth (SMD = 0.159), and embryo transfer number (SMD = 0.167) fall between 0.1 and 0.2, they remain below the conventional threshold of 0.25 for substantial imbalance, suggesting an acceptable balance (22). Results of the logistic regression analyses then indicated that higher dose of Crinone (180 vs. 90mg) was associated with all three pregnancy outcomes (CPR-S: OR = 2.17, 95% CI: 1.38–3.43, p < 0.001; CPR-H: OR = 2.58, 95% CI: 1.62–4.12, p < 0.001; LBR: OR = 2.10; 95% CI:1.31–3.38, p = 0.002) (**Table 5**).

**Table 5:** Association of vaginal progesterone gel dose and clinical pregnancy outcomes, with adjustment by IPTW.

Outcomes	OR (95%CI)	*P*-value
CPR-S	2.17 (1.38–3.43)	<0.001^*^
CPR-H	2.58 (1.62–4.12)	<0.001^*^
LBR	2.10 (1.31–3.38)	0.002^*^

IPTW, inverse probability of treatment weighting; OR, odds ratio; CI, confidence interval; CPR, clinical pregnancy rate; S, sac; H, heartbeat; LBR, live birth rate.

*P < 0.05 indicates statistical significance.

## Discussion

This study examined the impact of serum progesterone levels on the day of FET and the dose of vaginal progesterone gel (Crinone) used during LPS on clinical pregnancy outcomes. First, serum progesterone levels measured on the day of FET demonstrated poor predictive performance for pregnancy outcomes, as reflected by low AUC values in ROC analyses. Consistently, multivariable analyses showed no significant association between serum progesterone levels on FET day and clinical pregnancy outcomes, including CPR-S, CPR-H, or LBR. Second, in contrast to serum progesterone monitoring, the dose of vaginal progesterone gel appeared to have greater clinical relevance. After accounting for baseline differences using IPTW, a higher Crinone dose was associated with improved early clinical pregnancy outcomes (CPR-S and CPR-H). Although no significant association with LBR was observed in the multivariable model, an association emerged after IPTW adjustment and should be interpreted with caution. These findings suggest that, within AC-FET protocols using vaginal progesterone gel for early luteal phase support, the observed association between progesterone gel dose and pregnancy outcomes is worthy of further validation in prospective studies.

In this cohort of patients using vaginal progesterone gel for early luteal phase support, serum progesterone levels measured on the day of frozen embryo transfer were not significantly associated with pregnancy outcomes. The clinical significance of serum progesterone levels around the day of FET in patients undergoing artificial endometrial preparation remains an important area of investigation (6, 8, 9). A recent study reported that a serum progesterone level below 5.65 ng/ml on the day of FET was significantly associated with a lower ongoing pregnancy rate and LBR in women receiving oral dydrogesterone and intramuscular progesterone supplementation (23). Another study found an association between early luteal serum progesterone level (>252 nmol/l) and higher live births for FET using oral dydrogesterone (24). Furthermore, an earlier study demonstrated that a serum progesterone level < 9.2 ng/ml on the day of FET was associated with a lower ongoing pregnancy rate in women receiving vaginal micronized progesterone (25). In contrast, some studies found no correlation between serum progesterone levels and pregnancy outcomes, particularly those involving vaginal progesterone protocols (26, 27). For example, Zhu et al. found no correlation between serum progesterone and LBR in women receiving synchronized day 3 embryos or day 5 blastocysts following AC with a combination of oral dydrogesterone and vaginal micronized progesterone (27). In addition, Gonzalez-Foruria et al. (2023) did not find an association between pre-FET serum progesterone levels and LBR. Notably, they found that elevated progesterone levels (≥10.6 ng/ml) did not negatively impact LBR in women who received LPS with vaginal micronized progesterone alone or in combination with subcutaneous progesterone (26). Based on these prior studies, monitoring serum progesterone levels on the day of FET may be useful in certain clinical scenarios. However, these studies did not focus on patients using Crinone specifically. In this context, our findings add formulation-specific evidence suggesting that a single serum progesterone measurement on the day of FET may have limited predictive utility when Crinone is used for early luteal phase support.

The observed lack of association between serum progesterone levels and pregnancy outcomes may have several methodological explanations. Serum progesterone was measured at a single time point on the day of embryo transfer, which may not fully capture dynamic luteal phase exposure, particularly given the pulsatile secretion and variable absorption associated with vaginal progesterone formulations. This single measurement could have introduced exposure misclassification and reduced the ability to detect modest associations. In addition, the relatively low AUC values observed in the ROC analyses suggest limited discriminative performance, and small-to-moderate predictive effects cannot be excluded because of limited statistical power.

In our study, a higher dose of Crinone was associated with higher clinical pregnancy rates, and a potential association with LBR was observed after IPTW adjustment. A previous systematic review by Child et al. reported comparable clinical and ongoing pregnancy outcomes among commonly used vaginal progesterone preparations, including Crinone, across heterogeneous ART protocols (28). Importantly, those analyses primarily focused on formulation equivalence rather than dose–response effects within a specific treatment protocol. Therefore, these findings do not preclude the possibility of dose-related associations in specific, narrowly defined clinical settings. In contrast, the present study examined a specific AC-FET setting using vaginal progesterone gel for early luteal phase support, in which dose-related associations were observed after adjustment. Nevertheless, the observed association between a higher vaginal progesterone gel dose and pregnancy outcomes in our study aligns with previous findings, which reported significantly better reproductive outcomes with a higher dose of Crinone (90 mg twice daily) compared to a lower dose (90 mg once daily) in patients undergoing HRT-assisted FET (15). In their study, no additional progesterone supplementation was administered after FET, and serum progesterone levels were not reported (15).

A more recent prospective study, where patients were randomly assigned HRT consisting of vaginal progesterone capsules at 600 mg/day and 800 mg/day as part of endometrial preparation for day 5/6 blastocyst transfer (without progesterone rescue therapy or additional progesterone boosts), also reported that both the mean serum progesterone level on the blastocyst transfer day and the ongoing pregnancy rate was significantly higher in the 800 mg/day group (29). In our protocol, additional progesterone supplements administered via other routes were provided after FET. We found that when Crinone was used alone, serum progesterone level on the day of FET was not predictive of clinical pregnancy. This finding is supported by Enatsue et al. (2018), who compared the effectiveness of vaginal progesterone capsules administered at 900 and 1200 mg/day in women undergoing artificial endometrial preparation for day 5 FET (16). They reported significantly higher serum progesterone levels on the day of FET in the higher-dose group, and found that higher doses were associated with increased clinical pregnancy and live birth rates (16). They also found that for patients with a serum progesterone level was <9 ng/ml on the day of FET who were given additional oral progesterone supplementation as rescue therapy, serum progesterone level was not associated with clinical pregnancy (16). Collectively, prior studies suggest that higher doses of vaginal progesterone are generally associated with improved reproductive outcomes. However, a direct association between serum progesterone levels and pregnancy success remains inconsistent.

The effectiveness of rescue therapy with additional progesterone in patients with low serum progesterone levels around the day of FET has been examined in some studies, mostly in women undergoing vaginal progesterone-based LPS alone or in combination with intramuscular progesterone (30). A meta-analysis found that additional subcutaneous, intramuscular, or oral progesterone supplementation in those with low serum progesterone levels (cut-off range: 8.75–11 ng/ml) improved ongoing pregnancy rates and LBR to levels comparable to those in women with adequate serum progesterone (30). This is consistent with du Boulet et al. (2022), who also showed that additional oral dydrogesterone or subcutaneous progesterone could rescue ongoing pregnancy rates in women with low serum progesterone levels (<11 ng/ml) on the fourth day of vaginal progesterone-based LPS (31). In our study, Crinone was used for early luteal phase support prior to FET, and additional progesterone supplementation was provided after embryo transfer according to institutional practice; however, due to the retrospective design, the potential influence of the added progesterone on clinical outcomes could not be determined. Some previous studies have mainly focused on the potential impact of extreme serum progesterone levels rather than on a continuous dose–response relationship. For example, González-Foruria et al. reported that elevated serum progesterone levels (≥10.6 ng/mL) before FET did not negatively impact live birth rates, while Volovsky et al. demonstrated that very low progesterone levels (<5 ng/mL) on the day of FET were associated with reduced live birth rates. These findings suggest the presence of threshold effects at the extremes of serum progesterone exposure rather than a consistent linear association across the full range of values (26, 32).

Given the lack of data on early luteal phase hormone profiles, our finding of no association between serum progesterone levels on the day of FET and fertility outcomes in women undergoing AC-FET using Crinone for early LPS warrants further validation in prospective studies. Nevertheless, this study highlights that serum progesterone thresholds on the day of FET may not be a clinical concern for women using vaginal progesterone gel for early LPS. This may be explained by the distinct pharmacokinetics of vaginal progesterone, whereby serum concentrations may not accurately reflect the local biological effects of vaginal progesterone dosing.

A minor observation in our study was the slight difference in baseline endometrial thickness between the two groups, although this variation may be attributed to the limitations of the measurement method. Endometrial thickness was assessed via transabdominal ultrasound prior to embryo transfer, rather than transvaginal ultrasound. As a result, variations in bladder fullness among patients may have affected image quality. In addition, the assessments were performed by different operators, which may have introduced inter-observer variability. Nevertheless, all measured endometrial thicknesses were within the range considered suitable for embryo transfer.

This study has several limitations. First, the single-center, retrospective observational design may limit the generalizability of the findings and precludes causal inference. Although multivariable adjustment and inverse probability of treatment weighting were applied, residual confounding from unmeasured factors—such as provider-specific practice patterns, subtle clinical decision-making differences, or variability in post-transfer progesterone supplementation—cannot be excluded. Nevertheless, baseline characteristics including age, anti-Müllerian hormone levels, and causes of infertility were comparable between groups, suggesting that major sources of measured confounding were reasonably balanced.

Second, late luteal phase support regimens after embryo transfer were not standardized, and although these regimens were similarly distributed between groups, their independent effects on pregnancy outcomes could not be fully disentangled in this retrospective analysis.

Third, objective data on patient adherence to vaginal progesterone gel administration were unavailable. Variability in adherence may have contributed to inter-individual differences in effective progesterone exposure and may have attenuated the observed associations between progesterone dosing and pregnancy outcomes. This limitation may also affect generalizability to settings with different patient education or adherence patterns. Future prospective studies incorporating standardized luteal phase support protocols and adherence monitoring are therefore warranted to confirm these findings and to clarify the optimal dosing strategy of vaginal progesterone gel in artificial-cycle frozen embryo transfer.

## Conclusion

Serum progesterone levels measured on the day of FET showed limited predictive value for pregnancy outcomes when vaginal progesterone gel is used for early luteal phase support. In contrast, a higher Crinone dose (180 mg) was associated with higher CPR-S, CPR-H, and LBR compared with the lower dose (90 mg). However, these findings should be interpreted as hypothesis-generating rather than practice-changing, given the retrospective observational design. Prospective randomized controlled trials are therefore needed to determine the optimal dosing strategy of vaginal progesterone gel in artificial-cycle frozen embryo transfer.

## Data Availability

The raw data supporting the conclusions of this article will be made available by the authors, without undue reservation.
