# Supplementary material for: Serum progesterone and vaginal gel dose in artificial-cycle frozen embryo transfer: association with pregnancy outcome
Source: Front Endocrinol (Lausanne). 2026 Mar 5;17:1717071. doi: 10.3389/fendo.2026.1717071 (PMC12999430; doi:10.3389/fendo.2026.1717071)
Supplement: Supplementary file 1 [file Table1.docx]

**Supplementary Table S1. Distribution of late LPS regimens according to vaginal progesterone gel (Crinone) dose**

| **Late LPS regimen** | **90 mg (n = 108)** | **180 mg (n = 234)** |
| --- | --- | --- |
| **IM progesterone–based** |  |  |
| 3P | 81 (75.0%) | 77 (32.9%) |
| **Non-IM progesterone–based** |  |  |
| 4D | 27 (25.0%) | 73 (31.2%) |
| 2UP | 0 (0.0%) | 84 (35.9%) |

LPS, luteal phase support; IM, intramuscular; 3P, intramuscular progesterone injection, 50 mg administered every 3 days; 4D, oral dydrogesterone (Duphaston), four tablets per day; 2UP, oral micronized progesterone (Utrogestan), two capsules per day.
